# On avoided words, absent words, and their application to biological sequence analysis

**DOI:** 10.1186/s13015-017-0094-z

**Published:** 2017-03-14

**Authors:** Yannis Almirantis, Panagiotis Charalampopoulos, Jia Gao, Costas S. Iliopoulos, Manal Mohamed, Solon P. Pissis, Dimitris Polychronopoulos

**Affiliations:** 1 National Center for Scientific Research Demokritos, Neapoleos, 153 10 Athens, Greece; 2 Department of Informatics, King’s College London, The Strand, London, WC2R 2LS UK; 3 Computational Regulatory Genomics, MRC Clinical Sciences Centre (CSC), Du Cane Road, London, W12 0NN UK

**Keywords:** Avoided words, Underrepresented words, Absent words, Suffix tree, Conserved non-coding elements, Ultraconserved elements

## Abstract

**Background:**

The deviation of the observed frequency of a word *w* from its expected frequency in a given sequence *x* is used to determine whether or not the word is *avoided*. This concept is particularly useful in DNA linguistic analysis. The value of the deviation of *w*, denoted by $\textit{dev}(w)$, effectively characterises the extent of a word by its edge contrast in the context in which it occurs. A word *w* of length $k>2$ is a $\rho$-avoided word in *x* if $\textit{dev}(w) \le \rho$, for a given threshold $\rho < 0$. Notice that such a word may be completely *absent* from *x*. Hence, computing all such words naïvely can be a very time-consuming procedure, in particular for large *k*.

**Results:**

In this article, we propose an $\mathcal {O}(n)$-time and $\mathcal {O}(n)$-space algorithm to compute all $\rho$-avoided words of length *k* in a given sequence of length *n* over a fixed-sized alphabet. We also present a time-optimal $\mathcal {O}(\sigma n)$-time algorithm to compute all $\rho$-avoided words (of any length) in a sequence of length *n* over an integer alphabet of size $\sigma$. In addition, we provide a tight asymptotic upper bound for the number of $\rho$-avoided words over an integer alphabet and the expected length of the longest one. We make available an implementation of our algorithm. Experimental results, using both real and synthetic data, show the efficiency and applicability of our implementation in biological sequence analysis.

**Conclusions:**

The systematic search for avoided words is particularly useful for biological sequence analysis. We present a linear-time and linear-space algorithm for the computation of avoided words of length *k* in a given sequence *x*. We suggest a modification to this algorithm so that it computes all avoided words of *x*, irrespective of their length, within the same time complexity. We also present combinatorial results with regards to avoided words and absent words.

## Background

### Introduction

The one-to-one mapping of a DNA molecule to a sequence of letters suggests that DNA analysis can be modelled within the framework of formal language theory [1]. For example, a region within a DNA sequence can be considered as a “word” on a fixed-sized alphabet in which some of its natural aspects can be described by means of certain types of automata or grammars. However, a linguistic analysis of the DNA needs to take into account many distinctive physical and biological characteristics of such sequences: The genome consists of coding regions that encode for polypeptide chains associated with biological functions as well as a plethora of regulatory and potentially functional non-coding regions, identified through multiple alignment of genomes of several organisms, and termed conserved non-coding elements (CNEs). In addition, it contains large non-coding regions most of which are not linked to any particular function. All these genomic components appear to have many statistical features in common with natural languages [2].

A computational tool oriented towards the systematic search for avoided words is particularly useful for *in silico* genomic research analyses. The search for *absent words* is already undertaken in the recent past and several results exist on the application and computation of such words [3–6]. However, words which may be present in a genome or in genomic sequences of a specific role (e.g., protein coding segments, regulatory elements, conserved non-coding elements etc.) but they are strongly underrepresented—as we can estimate on the basis of the frequency of occurrence of their longest proper factors—may be of particular importance. They can be words of nucleotides which are hardly tolerated because they negatively influence the stability of the chromatin or, more generally, the functional genomic conformation; they can represent targets of restriction endonucleases which may be found in bacterial and viral genomes; or, more generally, they may be short genomic regions whose presence in wide parts of the genome are not tolerated for less known reasons. The understanding of such avoidances is becoming an interesting line of research (for recent studies, see [7, 8]).

On the other hand, short words of nucleotides may be systematically avoided in large genomic regions or whole genomes for entirely different reasons, i.e. just because they play important signaling roles which confine their appearance only in specific positions: consensus sequences for the initiation of gene transcription and of DNA replication are well-known such oligonucleotides. Other such cases may be insulators, sequences anchoring the chromatin on the nuclear envelope like lamina-associated domains, short sequences like dinucleotide repeat motifs with enhancer activity, and several other cases. Again, we cannot exclude that this area of research could lead to the identification of short sequences of regulatory activities still unknown.

Brendel et al. in [9] initiated research into the linguistics of nucleotide sequences that focuses on the concept of words in continuous languages—languages devoid of blanks—and introduced an operational definition of words. The authors suggested a method to measure, for each possible word *w* of length *k*, the deviation of its observed frequency from the expected frequency in a given sequence. The values of the deviation, denoted by $\textit{dev}(w)$, were then used to identify words that are avoided among all possible words of length *k*. The typical length of avoided (or of overabundant) words of the nucleotide language was found to range from 3 to 5 (tri- to pentamers). The statistical significance of the avoided words was shown to reflect their biological importance. This work, however, was based on the very limited sequence data available at the time: only DNA sequences from two viral and one bacterial genomes were considered. Also note that *k* might change when considering eukaryotic genomes, the complex dynamics and function of which might impose a more demanding analysis. The authors in [10–12] have studied the concept of unusual words—based on different definitions than the ones Brendel et al. use for expectation and variance—focusing on the factors of a string, whereas based on Brendel et al. definitions, we consider here *any* word over the alphabet.

### Our contributions

The computational problem can be described as follows. Given a sequence *x* of length *n*, an integer *k*, and a real number $\rho < 0$, compute the set of $\rho$-avoided words of length *k*, i.e. all words *w* of length *k* for which $\textit{dev}(w) \le \rho$. We call this set the $\rho$-avoided words of length *k* in *x*. Brendel et al. did not provide an efficient solution for this computation [9]. Notice that such a word may be completely absent from *x*. Hence the set of $\rho$-avoided words can be naïvely computed by considering all possible $\sigma ^k$ words, where $\sigma$ is the size of the alphabet.

Here we present an $\mathcal {O}(n)$-time and $\mathcal {O}(n)$-space algorithm for computing all $\rho$-avoided words of length *k* in a sequence of length *n* over a fixed-sized alphabet. For words over an integer alphabet of size $\sigma$, the algorithm requires time $\mathcal {O}(\sigma n)$, which is optimal for sufficiently large $\sigma$. We also present a time-optimal $\mathcal {O}(\sigma n)$-time algorithm to compute all $\rho$-avoided words (of any length) in a sequence of length *n* over an integer alphabet of size $\sigma$. We provide a tight asymptotic upper bound for the number of $\rho$-avoided words over an integer alphabet and the expected length of the longest one. We also prove that the same asymptotic upper bound is tight for the number of $\rho$-avoided words of fixed length when the alphabet is sufficiently large.

As shown subsequently, the set of absent $\rho$-avoided words is a subset of the set of minimal absent words of a word. Hence the tight asymptotic bounds for $\rho$-avoided words are based on the proof we provide for the tightness of the known asymptotic bound on minimal absent words and the tightness of this bound for minimal absent words of fixed length over sufficiently large alphabets.

We make available an open-source implementation of our algorithm. Experimental results, using both real and synthetic data, show its efficiency and applicability. Specifically, using our method we confirm that restriction endonucleases which target self-complementary sites are not found in eukaryotic sequences [8]. In addition, we apply our algorithm in the case of CNEs, which are classes of sequences whose functions in our genomes remain largely enigmatic [13, 14]. We observe interesting patterns of occurring avoided words within CNEs compared to CNE-like sequences (surrogates) that are in accordance with their distinct sequence characteristics which classify them from other non-functional sequences [15, 16].

A preliminary version of this article has appeared in [17].

## Methods

### Terminology and technical background

#### Definitions and notation

We begin with basic definitions and notation generally following [18]. Let $x=x[0]x[1] \cdots x[n-1]$ be a *word* of *length* $n=|x|$ over a finite ordered *alphabet* $\Sigma$ of fixed size $\sigma$, i.e. $\sigma = |\Sigma |=\mathcal {O}(1)$. We also consider the case of an *integer alphabet*; in this case each letter is replaced by its rank such that the resulting string consists of integers in the range $\{1,\ldots ,n\}$. For two positions *i* and *j* on *x*, we denote by $x[i \ldots j]=x[i]\cdots x[j]$ the *factor* (sometimes called *subword*) of *x* that starts at position *i* and ends at position *j* (it is empty if $j < i$), and by $\varepsilon$ the *empty word*, word of length 0. We recall that a prefix of *x* is a factor that starts at position 0 ($x[0\ldots j]$) and a suffix is a factor that ends at position $n-1$ ($x[i \ldots n-1]$), and that a factor of *x* is a *proper* factor if it is not *x* itself. A factor of *x* that is neither a prefix nor a suffix of *x* is called an $\textit{infix}$ of *x*. We say that *x* is *a power* of a word *y* if there exists a positive integer *k*, $k>1$, such that *x* is expressed as *k* consecutive concatenations of *y*; we denote that by $x=y^k$.

Let $w=w[0]w[1] \cdots w[m-1]$ be a word, $0<m\le n$. We say that there exists an *occurrence* of *w* in *x*, or, more simply, that *w* *occurs in* *x*, when *w* is a factor of *x*. Every occurrence of *w* can be characterised by a starting position in *x*. Thus we say that *w* occurs at the *starting position* *i* in *x* when $w=x[i \ldots i + m - 1]$. Further let *f*(*w*) denote the *observed frequency*, that is, the number of occurrences of a non-empty word *w* in word *x*. Note that overlapping occurrences are considered as distinct ones; e.g. $f(\texttt {TT})=2$ in $\texttt {TTT}$. If $f(w) = 0$ for some word *w*, then *w* is called *absent*, otherwise, *w* is called *occurring*.

By $f(w_p)$, $f(w_s)$, and $f(w_i)$ we denote the observed frequency of the longest proper prefix $w_p$, suffix $w_s$, and infix $w_i$ of *w* in *x*, respectively. We can now define the *expected frequency* of word *w*, $|w|>2$, in *x* as in Brendel et al. [9]:

1 $$\begin{aligned} E(w) = \frac{f(w_p) \times f(w_s)}{f(w_i)},\quad \text { if~ } f(w_i) >0; \text {~else~} E(w) = 0. \end{aligned}$$

The above definition can be explained intuitively as follows. Suppose we are given $f(w_p)$, $f(w_s)$, and $f(w_i)$. Given an occurrence of $w_i$ in *x*, the probability of it being preceded by *w*[0] is $\frac{f(w_p)}{f(w_i)}$ as *w*[0] precedes exactly $f(w_p)$ of the $f(w_i)$ occurrences of $w_i$. Similarly, this occurrence of $w_i$ is also an occurrence of $w_s$ with probability $\frac{f(w_s)}{f(w_i)}$. Although these two events are not always independent, the product $\frac{f(w_p)}{f(w_i)} \times \frac{f(w_s)}{f(w_i)}$ gives a good approximation of the probability that an occurrence of $w_i$ at position *j* implies an occurrence of *w* at position $j-1$. It can be seen then that by multiplying this product by the number of occurrences of $w_i$ we get the above formula for the expected frequency of *w*.

Moreover, to measure the deviation of the observed frequency of a word *w* from its expected frequency in *x*, we define the *deviation* ($\chi ^2$ test) of *w* as:

2 $$\begin{aligned} \textit{dev}(w) = \frac{f(w)-E(w)}{\max \{ \sqrt{E(w)}, 1\}}. \end{aligned}$$

For more details on the *biological* justification of these definitions see  [9].

Using the above definitions and a given threshold, we are in a position to classify a word *w* as either *avoided* or *common* in *x*. In particular, for a given threshold $\rho < 0$, a word *w* is called $\rho$-*avoided* if $\textit{dev}(w) \le \rho$. In this article, we consider the following computational problems.

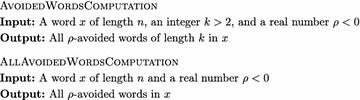



#### Suffix trees

In our algorithms, suffix trees are used extensively as computational tools. For a general introduction to suffix trees, see [18].

The *suffix tree* $\mathcal {T}(x)$ of a non-empty word *x* of length *n* is a compact trie representing all suffixes of *x*. The nodes of the trie which become nodes of the suffix tree are called *explicit* nodes, while the other nodes are called *implicit*. Each edge of the suffix tree can be viewed as an upward maximal path of implicit nodes starting with an explicit node. Moreover, each node belongs to a unique path of that kind. Then, each node of the trie can be represented in the suffix tree by the edge it belongs to and an index within the corresponding path.

We use $\mathcal {L}(v)$ to denote the *path-label* of a node *v*, i.e., the concatenation of the edge labels along the path from the root to *v*. We say that *v* is path-labelled $\mathcal {L}(v)$. Additionally, $\mathcal {D}(v)= |\mathcal {L}(v)|$ is used to denote the *word-depth* of node *v*. Node *v* is a *terminal* node, if and only if, $\mathcal {L}(v) = x[i \ldots n-1]$, $0 \le i < n$; here *v* is also labelled with index *i*. It should be clear that each occurring word *w* in *x* is uniquely represented by either an explicit or an implicit node of $\mathcal {T}(x)$. The *suffix-link* of a node *v* with path-label $\mathcal {L}(v)= \alpha y$ is a pointer to the node path-labelled *y*, where $\alpha \in \Sigma$ is a single letter and *y* is a word. The suffix-link of *v* exists if *v* is a non-root internal node of $\mathcal {T}(x)$. We denote by Child $(v,\alpha )$ the explicit node that is obtained from *v* by traversing the outgoing edge whose label starts with $\alpha \in \Sigma$.

In any standard implementation of the suffix tree, we assume that each node of the suffix tree is able to access its parent. Note that once $\mathcal {T}(x)$ is constructed, it can be traversed in a depth-first manner to compute the word-depth $\mathcal {D}(v)$ for each node *v*. Let *u* be the parent of *v*. Then the word-depth $\mathcal {D}(v)$ is computed by adding $\mathcal {D}(u)$ to the length of the label of edge (*u*, *v*). If *v* is the root then $\mathcal {D}(v) = 0$. Additionally, a depth-first traversal of $\mathcal {T}(x)$ allows us to count, for each node *v*, the number of terminal nodes in the subtree rooted at *v*, denoted by $\mathcal {C}(v)$, as follows. When internal node *v* is visited, $\mathcal {C}(v)$ is computed by adding up $\mathcal {C}(u)$ of all the nodes *u*, such that *u* is a child of *v*, and then $\mathcal {C}(v)$ is incremented by 1 if *v* itself is a terminal node. If a node *v* is a leaf then $\mathcal {C}(v) = 1$.

**Fig. 1:**
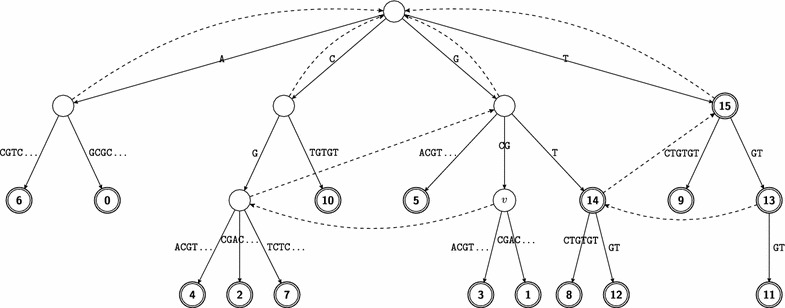
The suffix tree $\varvec{\mathcal {T}}\varvec{(x)}$ for $\varvec{x}= {{\mathbf {\mathtt{{AGCGCGACGTCTGTGT}}}}}$.* Double-lined* nodes represent terminal nodes labelled with the associated indices. The suffix-links for non-root internal nodes are* dashed*

##### *Example 1*

 Consider the word $x=\texttt {AGCGCGACGTCTGTGT}$. Fig. 1 represents the suffix tree $\mathcal {T}(x)$. Note that word $\texttt {GCG}$ is represented by the explicit internal node *v*; whereas word $\texttt {TCT}$ is represented by the implicit node along the edge connecting the node labelled 15 and the node labelled 9. Consider node *v* in $\mathcal {T}(x)$; we have that $\mathcal {L}(v) = \texttt {GCG}$, $\mathcal {D}(v) = 3$, and $\mathcal {C}(v)=2$.

### Tight bounds on minimal absent words

#### **Definition 1**

[4] An absent word *w* of *x* is *minimal* if and only if all proper factors of *w* occur in *x*.

We first show that the known asymptotic upper bound on the number of minimal absent words of a word is tight.

#### **Lemma 1**

[19]* The upper bound* $\mathcal {O}(\sigma n)$ *on the number of minimal absent words of a word of length* *n* *over an alphabet of size* $\sigma$ *is tight if* $2 \le \sigma \le n$.

#### *Proof*

 To prove that the bound is tight it suffices to construct a word with these many minimal absent words asymptotically.

Let $\Sigma =\{a_1,a_2\}$, i.e. $\sigma =2$, and consider the word $x=a_2 a_1^{n-2} a_2$ of length *n*. All words of the form $a_2 a_1^k a_2$ for $0 \le k \le n-3$ are minimal absent words in *x*. Hence *x* has at least $n-2=\Omega (n)$ minimal absent words.

Let $\Sigma =\{a_1,a_2,a_3,\ldots ,a_\sigma \}$ with $3 \le \sigma \le n$ and consider the word $x=a_2 a_1^k a_3 a_1^k a_4 a_1^k\cdots a_i a_1^k a_{i+1} \cdots a_{\sigma } a_1^k a_1^m$, where $k=\lfloor \frac{n}{\sigma -1}\rfloor -1$ and $m=n-(\sigma -1)(k+1)$. Note that *x* is of length *n*. Further note that $a_i a_1^j$ is a factor of *x*, for all $2 \le i \le \sigma$ and $0 \le j \le k$. Similarly, $a_1^j a_l$ is a factor of *x*, for all $3 \le l \le \sigma$ and $0 \le j \le k$. Thus all proper factors of all the words in the set $S=\{ a_i a_1^j a_l \, | \, 0 \le j \le k, \, 2 \le i \le \sigma , \, 3 \le l \le \sigma \}$ occur in *x*. However, the only words in *S* that occur in *x* are the ones of the form $a_i a_1^k a_{i+1}$, for $2 \le i < \sigma$. Hence *x* has at least $(\sigma -1)(\sigma -2)(k+1)-(\sigma -2)=(\sigma -1)(\sigma -2)\lfloor \frac{n}{\sigma -1}\rfloor -(\sigma -2)=\Omega (\sigma n)$ minimal absent words. $\square$

In the following lemma we show that, for sufficiently large alphabets, $\mathcal {O}(\sigma n)$ is a tight asymptotic bound for the number of minimal absent words of fixed length.

#### **Lemma 2**

*The upper bound* $\mathcal {O}(\sigma n)$ *on the number of minimal absent words of fixed length of a word of length* *n* *over an alphabet of size* $\sigma$ *is tight if* $\sqrt{n}+1 \le \sigma \le n$.

#### *Proof*

 Let $\Sigma =\{a_1, a_2, a_3,\ldots , a_\sigma \}$ be an alphabet of size $\sigma$. We will show that we can construct words of any length *n*, with $\sigma \le n \le \sigma (\sigma -1)$, that have $\Omega (\sigma n)$ minimal absent words of length 3.

We first construct the strings (blocks) $B_i= a_{i+1} a_i a_{i+2} a_i \cdots a_{i+j} a_i \cdots a_{\sigma } a_i$, for $1\le i \le \sigma -1$. Note that $|B_i|=2(\sigma -i)$ and that a letter $a_i$ occurs in $B_j$ if and only if $j \le i$. We then consider the word $x=B_1 B_2\cdots B_i\cdots B_{\sigma -1}$ which has length $|x|=\sum _{i=1}^{\sigma -1} 2(\sigma -i) = \sigma (\sigma -1)$.

Now consider any prefix *y* of *x* with $|y| > 2(\sigma -1)$. Then $y=B_1 B_2 \cdots B_{j-1} \overline{B_{j}}$, where $\overline{B_{j}}$ is a prefix of $B_{j}$ for some $j>1$. For any $i < j$ the words of length 3 with $a_i$ as the mid-letter that occur in *y* are the ones in the set $U_i=\{a_{\ell } a_i a_{\ell } \mid 1 \le \ell \le i-2\} \cup \{a_k a_i a_{k+1}\mid i+1 \le k \le \sigma -1 \} \cup \{a_{i-2} a_i a_{i-1}\}\cup \{a_{\sigma } a_i a_{i+2}\}$, with the last singleton not included if $i=j-1$ and $\overline{B_{j}}=\varepsilon$. We thus have $|U_i| \le \sigma$.

We notice that the strings of the form $a_k a_i$ for all $k \in P_i=\{1,2,\ldots ,\sigma \} \setminus \{i-1, i\}$ occur in *y* and similarly the strings of the form $a_i a_{\ell }$ for all $\ell \in S_i=\{1,2,\ldots ,\sigma \} \setminus \{i, i+1\}$ occur in *y*. Hence, all proper factors of all strings in $V_i=\{a_k a_i a_{\ell } \mid k \in P_i, \ell \in S_i\}$ occur in *y* and $|V_i|={(\sigma -2)}^2$. Then all the words in $M_i = V_i \setminus U_i$ are minimal absent words of *y* of length 3 with mid-letter $a_i$ and they are at least ${(\sigma -2)}^2-\sigma$. Now, since $|B_i| < 2 \sigma$ for all *i*, we have that $j > \frac{|y|}{2 \sigma }$. Hence $\sum _{i=1}^{j-1} |M_i| \ge ({(\sigma -2)}^2-\sigma ) \times \frac{|y|}{2 \sigma }$. Since the sets $M_i$ are pairwise disjoint it then follows that *y* has $\Omega (\sigma |y|)$ minimal absent words of length 3.

Hence, given an alphabet of size $\sigma$ we can construct words of any length *n*, such that $2\sigma < n \le \sigma (\sigma -1)$, that have $\Omega (\sigma n)$ minimal absent words of length 3.

Note that when $\sigma \le n \le 2 \sigma$ the example of $y=a_1 a_2 a_3 \cdots a_{\sigma }$ (possibly padded with $a_{\sigma }$’s) gives the desired result as at most $\sigma$ out of the ${\sigma }^2$ possible combinations $a_i a_j$ (of length 2) occur in *y*, while all proper factors of all such combinations occur in *y*.$\square$

### Useful properties of avoided words

In this section, we provide some useful insights of combinatorial nature which were not considered by Brendel et al. [9]. By the definition of $\rho$-avoided words it follows that a word *w* may be $\rho$-avoided even if it is absent from *x*. In other words, $\textit{dev}(w) \le \rho$ may hold for either $f(w) > 0$ (occurring) or $f(w) = 0$ (absent).

#### *Example 2*

 Consider again the word $x=\texttt {AGCGCGACGTCTGTGT}$, $k=3$, and $\rho =-0.4$.

- Word $w_1= \texttt {CGT}$, at position 7 of *x*, is an *occurring* $\rho$-avoided word:
  $$\begin{aligned} E(w_1) = 3\times 3/6 = 1.5,\text { } \textit{dev}(w_1) =(1-1.5)/\sqrt{1.5} = -0.408248. \end{aligned}$$
- Word $w_2 = \texttt {AGT}$ is an *absent* $\rho$-avoided word:
  $$\begin{aligned} E(w_2) = 1\times 3/6 = 0.5,\text { } \textit{dev}(w_2) =(0- 0.5)/1 = -0.5. \end{aligned}$$

This means that a naïve computation should consider *all* possible $\sigma ^k$ words. Then for each possible word *w*, the value of $\textit{dev}(w)$ can be computed via pattern matching on the suffix tree of *x*. In particular, we can search for the occurrences of *w*, $w_p$, $w_s$, and $w_i$ in *x* in time $\mathcal {O}(k)$ [18]. In order to avoid this inefficient computation, we exploit the following crucial lemmas.

#### **Lemma 3**

*Any absent* $\rho$-*avoided word* *w* *in* *x* *is a minimal absent word of* *x*.

#### *Proof*

 For *w* to be a $\rho$-avoided word it must hold that

$$\begin{aligned} \textit{dev}(w) = \frac{f(w)-E(w)}{\max \{ \sqrt{E(w)}, 1\}}\le \rho < 0. \end{aligned}$$

This implies that $f(w)-E(w)<0$, which in turn implies that $E(w)>0$ since $f(w) = 0$. From $E(w) = \frac{f(w_p) \times f(w_s)}{f(w_i)}>0$, we conclude that $f(w_p)>0$ and $f(w_s)>0$ must hold. Since $f(w) = 0$, $f(w_p)>0$, and $f(w_s)>0$, *w* is a minimal absent word of *x*: all proper factors of *w* occur in *x*. $\square$

#### **Lemma 4**

*Let* *w* *be a word occurring in* *x* *and* $\mathcal {T}(x)$ *be the suffix tree of* *x*.* Then, if* $w_p$ *is a path-label of an implicit node of* $\mathcal {T}(x)$, $\textit{dev}(w) \ge 0$.

#### *Proof*

 For any *w* that occurs in *x* it holds that $f(w_i) \ge f(w_s)$, which implies that $f(w_p) \ge \frac{f(w_p) \times f(w_s)}{f(w_i)} = E(w)$. Furthermore, by the definition of the suffix tree, if *w* occurs in *x* and $w_p$ is a path-label of an implicit node then $f(w_p) = f(w)$. It thus follows that $f(w) - E(w) = f(w_p) - E(w) \ge 0$, and since $\max \{1,\sqrt{E(w)}\} > 0$, the claim holds. $\square$

#### **Lemma 5**

*The number of* $\rho$-*avoided words of length* $k>2$ *in a word of length* *n* *over an alphabet of size* $\sigma$ *is* $\mathcal {O}(\sigma n)$;* in particular, this number is no more than* $(\sigma + 1) n - k + 1$.* The upper bound* $\mathcal {O}(\sigma n)$ *is tight if* $\sqrt{n}+1 \le \sigma \le n$.

#### *Proof*

 By Lemma 3, every $\rho$-avoided word is either occurring or a minimal absent word. It is known that the number of minimal absent words in a word of length *n* is smaller than or equal to $\sigma n$ [20]. Clearly, the occurring $\rho$-avoided words in a word of length *n* are at most $n - k + 1$. Therefore the number of $\rho$-avoided words of length *k* are no more than $(\sigma + 1) n - k + 1$. This implies that $\mathcal {O}(\sigma n)$ is an asymptotic upper bound. In the case of an alphabet of size $\sqrt{n}+1 \le \sigma \le n$, it follows from Lemma 2 that there exist words with $\Omega (\sigma n)$ minimal absent words of a fixed length $k>2$. Consider such a word *x*, the respective *k*, and some $\rho \ge - \frac{1}{n}$. Let *w* be any minimal absent word of *x*. We have that $f(w_p) \ge 1$, $f(w_s) \ge 1$, and $f(w_i) \le n$; and hence $E(w) \ge \frac{1}{n}$. Since $f(w)=0$, it follows that $\textit{dev}(w) \le - \frac{1}{n} \le \rho$. Thus, every minimal absent word of *x* is $\rho$-avoided, and since there are $\Omega (\sigma n)$ of them of length *k*, we conclude that $\mathcal {O}(\sigma n)$ is a tight asymptotic bound in this case. $\square$

### Avoided words algorithm

In this section, we present Algorithm AvoidedWords for computing all $\rho$-avoided words of length *k* in a given word *x*. The algorithm builds the suffix tree $\mathcal {T}(x)$ for word *x*, and then prepares $\mathcal {T}(x)$ to allow constant-time observed frequency queries. This is mainly achieved by counting the terminal nodes in the subtree rooted at node *v* for every node *v* of $\mathcal {T}(x)$. Additionally during this pre-processing, the algorithm computes the word-depth of *v* for every node *v* of $\mathcal {T}(x)$. By Lemma 3, $\rho$-avoided words are classified as either occurring or (minimal) absent, therefore Algorithm AvoidedWords calls Routines AbsentAvoidedWords and OccurringAvoidedWords to compute both classes of $\rho$-avoided words in *x*. The outline of Algorithm AvoidedWords is as follows.

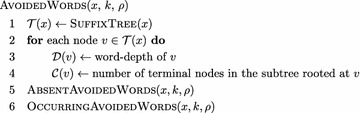



#### Computing absent avoided words

In Lemma 3, we showed that each absent $\rho$-avoided word is a minimal absent word. Thus, Routine AbsentAvoidedWords starts by computing all minimal absent words in *x*; this can be done in time and space $\mathcal {O}(n)$ for a fixed-sized alphabet or in time $\mathcal {O}(\sigma n)$ for integer alphabets [4, 5]. Let $< (i,j), \alpha>$ be a tuple representing a minimal absent word in *x*, where for some minimal absent word *w* of length $|w| > 2$, $w = x[i \ldots j]\alpha$, $\alpha \in \Sigma$; this representation is clearly unique.

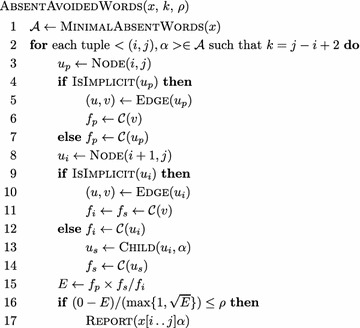



Intuitively, the idea is to check the length of every minimal absent word. If a tuple $< (i,j), \alpha>$ represents a minimal absent word *w* of length $k = j-i+2$, then the value of $\textit{dev}(w)$ is computed to determine whether *w* is an absent $\rho$-avoided word. Note that, if $w = x[i \ldots j]\alpha$ is a minimal absent word, then $w_p= x[i \ldots j]$, $w_i= x[i+1 \ldots j]$, and $w_s = x[i+1 \ldots j]\alpha$ occur in *x* by Definition 1. Thus, there are three (implicit or explicit) nodes in $\mathcal {T}(x)$ path-labelled $w_p$, $w_i$, and $w_s$, respectively.

The observed frequencies of $w_p$, $w_i$, and $w_s$ are already computed during the pre-processing of $\mathcal {T}(x)$. For an explicit node *v* of $\mathcal {T}(x)$, path-labelled $w'= x[i' \ldots j']$, the value $\mathcal {C}(v)$, which is the number of terminal nodes in the subtree rooted at *v*, is equal to the number of occurrences (observed frequency) of $w'$ in *x*. For an implicit node along the edge (*u*, *v*) path-labelled $w''$, the number of occurrences of $w''$ is equal to $\mathcal {C}(v)$ (and not $\mathcal {C}(u)$). The implementation of this procedure is given in Routine AbsentAvoidedWords.

#### Computing occurring avoided words

Lemma 4 suggests that for each occurring $\rho$-avoided word *w*, $w_p$ is a path-label of an explicit node *v* of $\mathcal {T}(x)$. Thus, for each internal node *v* such that $\mathcal {D}(v)= k-1$ and $\mathcal {L}(v)= w_p$, Routine OccurringAvoidedWords computes $\textit{dev}(w)$, where $w =w_p \alpha$, $\alpha \in \Sigma$, is a path-label of a child (explicit or implicit) node of *v*. Note that if $w_p$ is a path-label of an explicit node *v* then $w_i$ is a path-label of an explicit node *u* of $\mathcal {T}(x)$; node *u* is well-defined and it is the node pointed at by the suffix-link of *v*. The implementation of this procedure is given in Routine OccurringAvoidedWords.

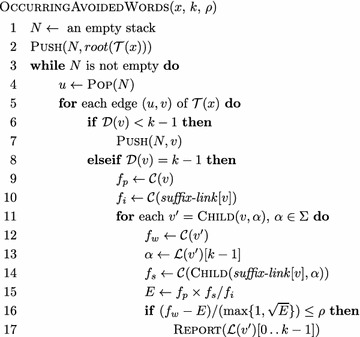



#### Analysis of the algorithm

##### **Lemma 6**

*Given a word* *x*,* an integer* $k>2$,* and a real number* $\rho < 0$,* Algorithm* AvoidedWords *computes all* $\rho$-*avoided words of length* *k* in *x*.

##### *Proof*

By definition, a $\rho$-avoided word *w* is either an absent $\rho$-avoided word or an occurring one. Hence, the proof of correctness relies on Lemmas 3 and 4. First, Lemma 3 indicates that an absent $\rho$-avoided word in *x* is necessarily a minimal absent word. Routine AbsentAvoidedWords considers each minimal absent word *w* and verifies if *w* is a $\rho$-avoided word of length *k*.

Second, Lemma 4 indicates that for each occurring $\rho$-avoided word *w*, $w_p$ is a path-label of an explicit node *v* of $\mathcal {T}(x)$. Routine OccurringAvoidedWords considers every child of each such node of word-depth *k*, and verifies if its path-label is a $\rho$-avoided word. $\square$

##### **Lemma 7**

*Given a word* *x* *of length* *n* *over a fixed-sized alphabet, an integer* $k>2$,* and a real number* $\rho < 0$,* Algorithm* AvoidedWords *requires time and space* $\mathcal {O}(n)$;* for integer alphabets, it requires time* $\mathcal {O}(\sigma n)$.

##### *Proof*

 Constructing the suffix tree $\mathcal {T}(x)$ of the input word *x* takes time and space $\mathcal {O}(n)$ for a word over a fixed-sized alphabet [18]. Once the suffix tree is constructed, computing arrays $\mathcal {D}$ and $\mathcal {C}$ by traversing $\mathcal {T}(x)$ requires time and space $\mathcal {O}(n)$. Note that the path-labels of the nodes of $\mathcal {T}(x)$ can by implemented in time and space $\mathcal {O}(n)$ as follows: traverse the suffix tree to compute for each node *v* the smallest index *i* of the terminal nodes of the subtree rooted at *v*. Then $\mathcal {L}(v) = x[i \ldots i+\mathcal {D}(v)-1]$.

Next, Routine AbsentAvoidedWords requires time $\mathcal {O}(n)$. It starts by computing all minimal absent words of *x*, which can be achieved in time and space $\mathcal {O}(n)$ over a fixed-sized alphabet [4, 5]. The rest of the procedure deals with checking each of the $\mathcal {O}(n)$ minimal absent words of length *k*. Checking each minimal absent word *w* to determine whether it is a $\rho$-avoided word or not requires time $\mathcal {O}(1)$. In particular, an $\mathcal {O}(n)$-time pre-processing of $\mathcal {T}(x)$ allows the retrieval of the (implicit or explicit) node in $\mathcal {T}(x)$ corresponding to the longest proper prefix of *w* in time $\mathcal {O}(1)$ [21]. Finally, Routine OccurringAvoidedWords requires time $\mathcal {O}(n)$. It traverses the suffix tree $\mathcal {T}(x)$ to consider all explicit nodes of word-depth $k-1$. Then for each such node, the procedure checks every (explicit or implicit) child of word-depth *k*. The total number of these children is at most $n-k+1$. For every child node, the procedure checks whether its path-label is a $\rho$-avoided word in time $\mathcal {O}(1)$ via the use of suffix-links.

For integer alphabets, the suffix tree can be constructed in time $$\mathcal {O}(n)$$ [22] and all minimal absent words can be computed in time $$\mathcal {O}(\sigma n)$$ [4, 5]. The efficiency of Algorithm AvoidedWords is then limited by the total number of words to be considered, which, by Lemma 5, is $$\mathcal {O}(\sigma n)$$. Note that for integers alphabets, a batch of $$q $$ Child $$(v,\alpha) $$ queries can be answered off-line in time $$\mathcal{O}(n+q)$$ with the aid of radix sort (in Routine AbsentAvoidedWords) or on-line in time $$\mathcal{O}(q \log \sigma) $$ (in Routine OccurringAvoidedWords).$$\square $$

Lemmas 5, 6 and 7 imply the first result of this article.

##### **Theorem 1**

*Algorithm* AvoidedWords *solves Problem* AvoidedWordsComputation *in time and space* $\mathcal {O}(n)$.* For integer alphabets, the algorithm solves the problem in time* $\mathcal {O}(\sigma n)$;* this is time-optimal if* $\sqrt{n}+1 \le \sigma \le n$.

### Optimal computation of all* ρ*-avoided words

Although the biological motivation is yet to be shown for this, we present here how we can modify Algorithm AvoidedWords so that it computes *all* $\rho$-avoided words (of all lengths) in a given word *x* of length *n* over an integer alphabet of size $\sigma$ in time $\mathcal {O}(\sigma n)$. We further show that this algorithm (AllAvoidedWords) is in fact time-optimal.

Based on Lemma 1 and similarly to the proof of Lemma 5 we obtain the following result.

#### **Lemma 8**

*The number of* $\rho$-*avoided words in a word of length* *n* *over an alphabet of size* $2 \le \sigma \le n$ *is* $\mathcal {O}(\sigma n)$ *and this bound is tight*.

It is clear that if we just remove the condition on the length of each minimal absent word in Line 2 of AbsentAvoidedWords we then compute all absent $\rho$-avoided words in time $\mathcal {O}(\sigma n)$. In order to compute all occurring $\rho$-avoided words in *x* it suffices by Lemma 4 to investigate the children of explicit nodes. We can thus traverse the suffix tree $\mathcal {T}(x)$ and for each explicit internal node, check for all of its children (explicit or implicit) whether their path-label is a $\rho$-avoided word. We can do this in $\mathcal {O}(1)$ time as described. The total number of these children is at most $2n-1$, as this is the bound on the number of edges of $\mathcal {T}(x)$ [18]. This modified algorithm is clearly time-optimal for fixed-sized alphabets as it then runs in time $\mathcal {O}(n)$. The time optimality for integer alphabets follows directly from Lemma 8. Hence we obtain the second result of this article.

#### **Theorem 2**

*Algorithm* AllAvoidedWords *solves Problem* AllAvoidedWordsComputation *in time* $\mathcal {O}(\sigma n)$.* This is time-optimal if* $2 \le \sigma \le n$.

#### *Remark 1*

In [23], it is shown that all $|\mathcal{A}|$ minimal absent words of a word *x* of length *n* over an integer alphabet can be computed in time $\mathcal{O}(n+|\mathcal{A}|)$ and space $\mathcal {O}(n)$. Computing minimal absent words and checking for each of them if it is an avoided word is the bottleneck for algorithms AvoidedWords and AllAvoidedWords. The result of [23] implies that for a word *x* of length *n* over an integer alphabet we can make both algorithms to require time $\mathcal{O}(n+|\mathcal{A}|)$ and space $\mathcal {O}(n)$. We can do that by checking for each minimal absent word output by the algorithm whether it is avoided, instead of storing a representation of them and then making the check.

#### *Remark 2*

As the complexity of algorithms AvoidedWords and AllAvoidedWords does not depend on the value of $\rho$, one can use a negative $\rho$ close to 0, sort the output $\rho$-avoided words with respect to $\textit{dev}(w)$, and consider the extreme ones.

#### **Lemma 9**

*The expected length of the longest* $\rho$-*avoided word in a word* *x* *of length* *n* *over an alphabet* $\Sigma$ *of size* $\sigma >1$ *is* $\mathcal {O}(\log _{\sigma } n)$ *when the letters are independent and identically distributed random variables uniformly distributed over* $\Sigma$.

#### *Proof*

 By Lemma 4 the length of the longest occurring word is bounded above by the word-depth of the deepest internal explicit node in $\mathcal {T}(x)$ incremented by 1. We note that the greatest word-depth of an internal node corresponds to the longest repeated factor in word *x*. Moreover, for a word *w* to be a minimal absent word, $w_i$ must appear at least twice in *x* (in the occurrences of $w_p$ and $w_s$). Hence the length of the longest $\rho$-avoided word is bounded by the length of the longest repeated factor in *x* incremented by 2. The expected length of the longest repeated factor in a word is known to be $\mathcal {O}(\log _{\sigma } n)$ [24] and hence the lemma follows. $\square$

## Experimental results

Algorithm AvoidedWords was implemented as a program to compute the $\rho$-avoided words of length *k* in one or more input sequences; there is an option to run Algorithm AllAvoidedWords instead. The program was implemented in the C++ programming language and developed under GNU/Linux operating system. Our program makes use of the implementation of the compressed suffix tree available in the Succinct Data Structure Library [25]. The input parameters are a (Multi)FASTA file with the input sequence(s), an integer $k > 2$, and a real number $\rho < 0$. The output is a file with the set of $\rho$-avoided words of length *k* per input sequence. The implementation is distributed under the GNU General Public License, and it is available at http://github.com/solonas13/aw. The experiments were conducted on a Desktop PC using one core of Intel Core i5-4690 CPU at 3.50 GHz under GNU/Linux. The program was compiled with g++ version 4.8.4 at optimisation level 3 (−O3). We also implemented a brute-force approach for the computation of $\rho$-avoided words. We mainly used it to confirm the correctness of our implementation. Here we do not plot the results of the brute-force approach as it is easily understood that it is orders of magnitude slower than our approach.

**Fig. 2:**
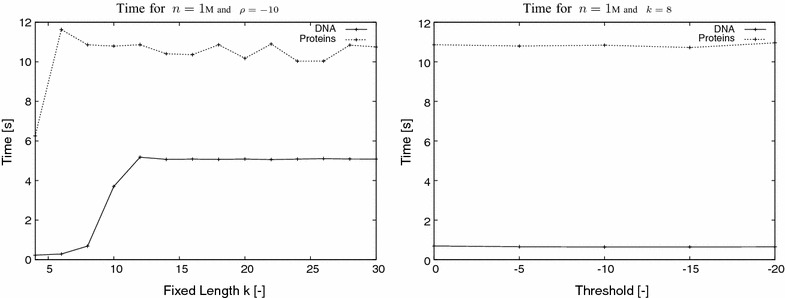
Experiment I. Elapsed time of Algorithm AvoidedWords using synthetic DNA ($\sigma =4$) and proteins ($\sigma =20$) data of length 1M for variable *k* and variable $\rho$

### Experiment I

To evaluate the time performance of our implementation, synthetic DNA ($\sigma =4$) and protein ($\sigma =20$) data were used. The input sequences were generated using a randomised script. In the first experiment, our task was to establish that the performance of the program does not essentially depend on *k* and $\rho$; i.e., the elapsed time of the program remains unchanged up to some constant with increasing values of *k* and decreasing values of $\rho$. As input datasets, for this experiment, we used a DNA and a protein sequence both of length 1M (1 Million letters). For each sequence we used different values of *k* and $\rho$. The results, for elapsed time are plotted in Fig. 2. It becomes evident from the results that the time performance of the program remains unchanged up to some constant. The longer time required for the protein sequences for some value of *k* is explained by the increased number of branching nodes in this depth in the corresponding suffix tree due to the size of the alphabet ($\sigma =20$). To confirm this we counted the number of nodes considered by the algorithm to compute the $\rho$-avoided words for $k=4$ and $\rho =-10$ for both sequences. The number of considered nodes for the DNA sequence was 260 whereas for the protein sequence it was 1,585,510.

**Fig. 3:**
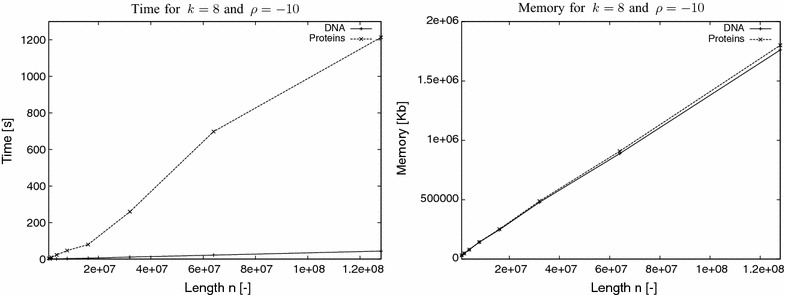
Experiment II. Elapsed time and peak memory usage of Algorithm AvoidedWords using synthetic DNA ($\sigma =4$) and proteins ($\sigma =20$) data of length 1–128M

### Experiment II

In the second experiment, our task was to establish the fact that the elapsed time and memory usage of the program grow linearly with *n*, the length of the input sequence. As input datasets, for this experiment, we used synthetic DNA and proteins sequences ranging from 1 to 128 M. For each sequence we used constant values for *k* and $\rho$: $k=8$ and $\rho =-10$. The results, for elapsed time and peak memory usage, are plotted in Fig. 3. It becomes evident from the results that the elapsed time and memory usage of the program grow linearly with *n*. The longer time required for the protein sequences compared to the DNA sequences for increasing *n* is explained by the increased number of branching nodes in this depth ($k=8$) in the corresponding suffix tree due to the size of the alphabet ($\sigma =20$). To confirm this we counted the number of nodes considered by the algorithm to compute the $\rho$-avoided words for $n=64$M for both the DNA and the protein sequence. The number of nodes for the DNA sequence was 69,392 whereas for the protein sequence it was 43,423,082.

**Fig. 4:**
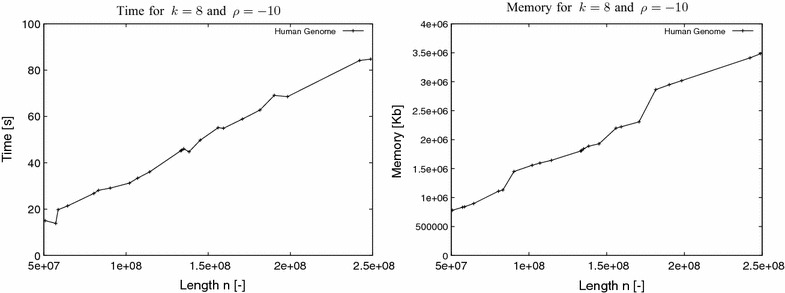
Experiment III. Elapsed time and peak memory usage of Algorithm AvoidedWords using all chromosomes of the human genome

### Experiment III

 In the next experiment, our task was to evaluate the time and memory performance of our implementation with real data. As input datasets, for this experiment, we used all chromosomes of the human genome. Their lengths range from around 46M (chromosome 21) to around 249M (chromosome 1). For each sequence we used $k=8$ and $\rho =-10$. The results, for elapsed time and peak memory usage, are plotted in Fig. 4. The results with real data confirm that the elapsed time and memory usage of the program grow linearly with *n*.

### Experiment IV

 In an experiment with a prokaryote, we computed the set of avoided words for $k=6$ (hexamers) and $\rho =-10$ in the complete genome of *Escherichia coli* and sorted the output in increasing order of their deviation. The most avoided words were extremely enriched in self-complementary (palindromic) hexamers. In particular, within the output of 28 avoided words, 23 were self-complementary; and the 17 most avoided ones were *all* self-complementary. For comparison, we computed the set of avoided words for $k=6$ and $\rho =-10$ from an eukaryotic sequence: a segment of the human chromosome 21 (its leftmost segment devoid of N’s) equal to the length of the *E. coli* genome. In the output of 10 avoided words, no self-complementary hexamer was found. Our results confirm that the restriction endonucleases which target self-complementary sites are not found in eukaryotic sequences [8].

**Table 1:** The number of avoided words, for $k=10$ and $\rho =-2$, for each concatenate of surrogates (Row 1); the number of avoided words of the corresponding CNE dataset (Row 2); and their ratio (Row 3)

	CNEs 75–80	CNEs 80–85	CNEs 85–90	CNEs 90–95	CNEs 95–100	Mammalian	Amniotic
Surr.	1658	810	445	256	429	29,677	6043
CNE	514	153	51	40	45	2821	623
Ratio	3.23	5.29	8.73	6.40	9.53	10.52	9.70

**Table 2:** The number of avoided words, for $k>2$ and $\rho =-2$, for each concatenate of surrogates (Row 1); the number of avoided words of the corresponding CNE dataset (Row 2); and their ratio (Row 3)

	CNEs 75–80	CNEs 80–85	CNEs 85–90	CNEs 90–95	CNEs 95–100	Mammalian	Amniotic
Surr.	10,734	7202	5351	3849	4540	112,181	22,595
CNE	3207	1847	1296	1043	1030	17,685	3635
Ratio	3.35	3.90	4.13	3.69	4.41	6.34	6.22

### Experiment V

 Then, we proceeded to the examination of several collections of CNEs obtained through multiple sequence alignment between the human and other genomes. The detailed description of how those CNEs were identified could be found in [15]. For each CNE of these datasets, a sequence stretch (surrogate sequence) of non-coding DNA of equal length and equal GC content was taken at random from the repeat-masked human genome. The CNEs of each collection were concatenated into a single long sequence and the same procedure was followed for the corresponding surrogates. Seven CNEs concatenates and the corresponding surrogate datasets have been formed and used in this experiment. We have determined through the proposed algorithm the avoided words for $k=10$ (decamers) and $\rho =-2$ for these fourteen datasets and the results are presented in Table 1. In Table 2, we show likewise for $k>2$ (all avoided words) and $\rho =-2$.

The first five CNEs collections have been composed through multiple sequence alignment of the same set of genomes and they differ only in the thresholds of sequence similarity applied between the considered genomes: from 75 to 80 (the least conserved CNEs, which thus are expected to serve less demanding functional roles) to 95–100 which represent the extremely conserved non-coding elements (UCNEs or CNEs 95–100) [15]. The remaining two collections have been composed under different constraints and have been derived after alignment of genomes belonging to the *Mammalian* and *Amniotic* groups. In Tables 1 and 2, the last line shows the ratios formed by the numbers of avoided words of each concatenate of surrogates divided by the numbers of avoided words of the corresponding CNE dataset.

Two immediate results stem from inspection of Tables 1 and 2:

1. In all cases, the number of avoided words from the non-functional (surrogate) concatenate of sequences far exceeds the corresponding number derived from the corresponding CNE dataset.
2. In the case of datasets with increasing degree of similarity between aligned genomes (from 75–80 to 95–100) the ratios of the numbers of avoided words show a clear increasing trend.

Both these findings can be understood on the basis of the difference in functionality, and thus tolerance to mutations, between CNE and surrogate datasets. One particularly frequent source of mutations is the slippage error during DNA replication; see e.g. reference [26]. Within a genomic sequence, this phenomenon causes the generation and increase in length, during evolutionary time, of polypyrimidine and polypurine nucleotide tracts. The expansion of those tracts is impeded at a considerable degree in the case of sequences which serve a functional role (as CNEs do) due to several constraints. On the other hand, in non-functional regions (as our surrogates mostly are) this procedure ceases to be tolerated only when it reaches to the formation of a polypyrimidine/polypurine tract with length affecting the proper folding or other structural features of the chromatin. Then, selection eliminates it, while its longer proper factors are tolerated in sufficient numbers within the sequence, thus resulting to an avoided word. In support of this explanation is the observation that all lists of avoided words found by our algorithm in concatenates of surrogates exhibit a considerable enrichment in oligopurines and oligopyrimidines. Taking at random some examples, for $k=10$, we notice: AAAAAAAAAT, AAAAAACCAC, ACAAAAAAAA, CTCCTCTTTT, etc.

Our second observation, i.e. the positive correlation between (1) the paucity of avoided decamers in CNEs collections and (2) the similarity thresholds used for their identification comes in accordance with the above argument. CNEs extracted under a stricter requirement of sequence similarity between evolutionary distant species are CNEs whose functionality is less tolerant to alterations due to random mutations in general. Hence, they also tolerate less the propagation within their sequence of parasite polypyrimidine/polypurine tracts too.

## Conclusions

We presented an $\mathcal {O}(n)$-time and $\mathcal {O}(n)$-space algorithm to compute all $\rho$-avoided words of length *k* in a sequence of length *n* over a fixed-sized alphabet. For integer alphabets, our algorithm runs in time $\mathcal {O}(\sigma n)$ and is optimal for a sufficiently large alphabet of size $\sigma$. We also presented a time-optimal $\mathcal {O}(\sigma n)$-time algorithm to compute all $\rho$-avoided words (of any length) in a sequence of length *n* over an integer alphabet. Moreover, we provided a tight asymptotic upper bound for the number of $\rho$-avoided words over an integer alphabet and the expected length of the longest one.

In the process, we showed that the known asymptotic upper bound on the number of minimal absent words of a sequence is tight for integer alphabets. We also showed that the same asymptotic bound is tight for the number of minimal absent words of a fixed length if the alphabet is sufficiently large.

Finally, we made available an implementation of our algorithm. Experimental results, using both real and synthetic data, show its efficiency and applicability in biological sequence analysis.
